# Infracentimetric HER-2 positive breast tumours—review of the literature

**DOI:** 10.3332/ecancer.2015.593

**Published:** 2015-11-18

**Authors:** Danilo da Fonseca Reis Silva, Joana M Ribeiro

**Affiliations:** 1 Instituto do Câncer do Estado de São Paulo – ICESP – Faculdade de Medicina da Universidade de São Paulo, Av. Doutor Arnaldo, 251 - Cerqueira César, São Paulo - SP 01246-000, Brazil; 2 Breast Unit, Champalimaud Clinical Centre, Av. de Brasília, s/n, Lisbon 1400-038, Portugal

**Keywords:** breast cancer, infracentimetric, HER-2 positive breast cancer

## Abstract

Breast cancer is the most common malignant neoplasm in the world among women. As a result of the dissemination of population screening programmes, about half of non-metastatic breast cancers are now diagnosed at stage I. 10–15% of T1abN0 tumours over-express human epidermal growth factor (HER-2). These tumours have a globally excellent prognosis, however, treatment with chemotherapy and/or targeted therapy may further improve outcomes in selected cases. In this article, we will review studies with information on prognosis and benefit of adjuvant therapy for T1abN0 HER-2+ breast cancer.

## 1 Introduction

Breast cancer is the most common malignant neoplasm in the world among women, representing approximately 25.2% of new cases of cancer [1]. Data from SEER (the Surveillance, Epidemiology, and End Results Programme) indicate that approximately 12.5% of American women will have this diagnosis at some point in their lives [2]. In the last few decades, there has been an increase in the proportion of cases diagnosed at earlier stages, such that stage I tumours currently represent nearly half of the cases of non-metastatic disease [3]. According to SEER between 1990 and 1998, there was a 15% increase in the incidence of T1 tumours (0–2 cm) in the United States (from 143.5–163.5 per 100,000) [4], which is mainly attributed to the detection of non-palpable tumours through the dissemination of population screening programmes with mammography [3, 4].

The overexpression of Human epidermal growth factor-2 (HER-2+) is found in about 20% of breast tumours and is associated with worse survival rates, especially in the population with positive lymph nodes [5–7]. Among infracentimetric tumours (T1ab), only 10–15% are HER-2 positive [8–10], which may reflect the increased aggressiveness of these tumours, as they are frequently diagnosed at more advanced stages.

Globally, it is considered that patients with breast cancer of less than 1 cm have a good prognosis, however, until recently most series did not contain information regarding HER-2 status and the adjuvant therapeutic clinical trials focused on patients with HER-2+ breast tumours excluded those patients [11–15]. In this article, we will review the retrospective and prospective studies as well as the meta-analyses with information relating to the prognosis and therapeutic impact of chemotherapy and/or anti-HER-2 targeted therapy among patients with T1ab tumours.

## 2 Breast carcinoma T1ab HER-2+: information on prognosis

Most studies focused on patients with T1ab breast cancer without lymph node involvement have suggested that these patients, overall, have an excellent prognosis, having a breast cancer specific survival at ten years greater than 95% [16–18]. However, not all studies were consistent in terms of the reported survival rates [19–21]. The discrepancy observed is probably related to the intrinsic limitations and heterogeneity of the studies which assessed this issue, namely: heterogeneity of the adjuvant systemic treatments used, different techniques and definitions of HER-2 status, reduced sample sizes, and different methodologies used (for example, most studies used different metrics for survival—while some reported disease-free survival (DFS), others recurrence-free survival (RFS)—similar to DFS, but excluding new primary cancers whether of the ipsilateral or the contralateral, while others used breast cancer specific survival (BCSS) and/or distant DFS (DDFS) (Table 1) [8, 9, 20–24].

One of the first studies which analysed the prognosis for infracentimetric tumours assessed a population of 242 patients with lymph node negative breast cancer, of which 19% were HER-2+ tumours, versus 83% with HER-2 negative (HER-2-). The RFS at two years was 94% versus 83% for HER-2- and HER-2+ tumours respectively (p < 0.05). In this study, for the population of infracentimetric tumours, the HER-2+ status was an independent variable for prognosis with a relative risk of recurrence of 4.6 (confidence interval (CI) 95% 1.0–20.6) and death of 11.1 (CI 95% 1.0–122.8) when comparing HER-2+ with HER-2- tumours [23]. Likewise, in 2003, a study by a Finnish group included 852 stage I patients–36.7% of these T1ab–demonstrating a relative risk of 2.6 in DDFS at nine years for HER-2+ tumours when compared with HER-2- tumours (89% versus 73%, CI 95% 1.1–6.2; p < 0.01) [21]. Chia *et al* analysed 2026 patients with lymph node negative breast cancer, of which 10.2% were HER-2+. In this cohort, the RFS at 10-years was lower for HER-2+ tumours when compared to HER-2- tumours (65.9% versus 75.5% respectively p = 0.01). Nevertheless, the number of infracentimetric HER-2+ tumours in this cohort was small (n = 21). In 2009, Gonzalez *et al* published a retrospective series of 965 patients with T1ab tumours treated at the MD Anderson Cancer Centre from 1990–2002 whose patients did not receive treatment with adjuvant chemotherapy or trastuzumab. In this study, HER-2+ status was also strongly associated with a worse RFS at five years (77.1% versus 93.7% p < 0.001) [9]. In addition, this study also had a validation cohort with 350 tumours from the Institute Jules Bordet and Leoben General Hospital which also suggested a lower RFS at five years for the HER-2+ tumours group (87% versus 97%; p = .043) [9]. At the end of the same year, Curigliano and collaborators published a series of 2130 T1ab breast cancer patients—150 (7%) with HER-2+—looked at the prognostic role of HER-2 status for small tumours [25]. After 4.6 years of follow-up, HER-2 overexpression was associated with a relative risk of 2.4 (CI 95%, 0.9–6.5; p 0.09) for DFS at five years. Recently Vaz-Luis *et al* in a series with more than 4000 patients with T1abN0 tumours, 520 of which with HER-2+ disease, reported distant relapse at 5 years for patients not treated with chemotherapy or trastuzumab that did not exceed 7% [10]. Fehrenbacher et al examined the outcomes of 234 patients with HER-2+ T1ab tumours diagnosed between 2000 and 2006 and similarly the prognosis for the patients not treated with chemotherapy or trastuzumab (n = 171) was, in general, excellent, with rates of recurrence (distant and local recurrence free interval) in all subgroups of less than 10% at 5 years [22]. Tables 1 and 2 summarise the survival estimates of the different studies.

### 2.1 Impact of hormone receptors on the prognosis for T1ab HER-2+ tumours

The impact of the hormone receptors (HR) in the HER-2+ population has not yet been completely clarified. In the National Comprehensive Cancer Network (NCCN) series, T1b tumours had an invasive disease-free survival (IDFS) at 5 years of 68% (CI 95% 40–86%) versus 86% (CI 95% 76–92%) for HR+ T1b tumours [10]. In this same series the estimates of DDFS at 5 years were similar in the two groups, 94% (CI 95%, 63–99%) for patients with HR Negative (HR-) T1b tumours not treated with chemotherapy/trastuzumab and 94% (CI 95%, 86–98%) for patients with HR+ tumours. Of note, the wide confidence intervals reported in a cohort study reported by Chia *et al* on 117 patients with stage I HER-2+ breast cancer, the HR+ subgroup showed a higher RFS at 10 years (77.5% versus 68.3%) [8]. Finally, the series published by Fehrenbacher covered a population of 234 patients of which 73.1% did not receive chemotherapy or trastuzumab, among those, the 138 (59%) patients with HR+ tumours treated had a recurrence free interval (RFI) similar to the 96 (41%) of patients with HR- tumours (94.9% versus 95.1%, respectively). Of note, 116 (50%) patients had T1a tumours, which may have made the difference between the subgroups undetectable. In addition, the RFI for the HR- subgroup, when both the treated and untreated patients are analysed, was numerically lower (92.9% versus 95%) [22]. Globally these data indicate that the hormonal status may have a role in the prognosis for infracentimetric HER-2+ tumours, with some but not all series indicating unfavourable outcomes in HR- patients at five years.

### 2.2 Impact of size on the prognosis for T1ab HER-2+ tumours

In the article mentioned previously by Fehrenbacher, the invasive RFI at 5 years was 97% (CI 95%, 90.9–99.0%), 91.9% (CI 95%, 81.5–96.6%), and 89.4% (CI 95%, 70.6–96.5%) for T1a, T1b, and T1b = 1cm tumours, respectively [22]. These data are in line with the findings of Verscraegen *et al* who reported a logarithmic linear relationship between tumour size and death due to breast cancer, especially for small tumours [26]. The Vaz-Luis *et al* series also suggested that the tumour size can influence the risk of recurrence [10].

## 3 Impact of treatment with chemotherapy and/or trastuzumab on T1ab HER-2+ tumours

Data derived from retrospective studies seems to demonstrate a benefit from adjuvant treatment based on trastuzumab in this population. The NCCN series examined survival estimates in treated and non-treated groups with chemotherapy and trastuzumab. Although the design of the study does not permit formal comparisons between the groups, the patients with T1a tumours who received treatment (chemotherapy and/or trastuzumab), had IDFS values at five years of 89–100% and DDFS values of 100%. For the T1b tumours, the IDFS was 90–94% and the DDFS was 94–96% for the treated patients versus an IDFS at five years of 68–86% and a DDFS of 94% for the untreated patients [10] (Table 2).

A single-centre study at the Memorial Sloan-Kettering Cancer Centre reported a three-years DDFS of 97% (CI 95%, 92–100%) in a group of 45 patients not treated with trastuzumab and of 100% in a group of 54 patients treated with trastuzumab [27, 28].

In addition, another retrospective study of the Institut Curie (Paris, France) included 97 patients with tumours smaller than or equal to 1 cm between 2002 and 2008, all without lymph node involvement. Adjuvant trastuzumab based therapy was given to patients considered to have a high risk profile—HR-, high grade or high mitotic index according to institutional criteria. Of the 97 T1abN0 HER-2+ patients, 41 (42%) received trastuzumab and 93% of these also received chemotherapy. None of the patients treated with anti-HER-2 therapy had a recurrence, while 9% of those who did not receive adjuvant treatment with trastuzumab had recurrence (5/56–4 of the recurrences in the subgroup with negative hormone receptors) (p = 0.11) [29].

A meta-analysis presented at the ASCO Annual Meeting 2014 was performed to estimate overall survival (OS) and DFS with the addition of adjuvant trastuzumab for tumours ≤ 2 cm, independently of lymph node status, using five randomized clinical trials published between 2004 and 2013. Of the 11,200 patients randomised in these five studies, 4220 fulfilled the criteria for tumour size (2588 were randomized to receive trastuzumab and 1632 not). The majority of the study population was composed of T1c tumours and had lymph node involvement. Two cohorts were analysed according to HR status. The two cohorts showed a reduction in relative risk of 30% (HR = 0.7, CI 95% 0.58–0.85) for the group receiving trastuzumab. The HR+ patient cohort showed a cumulative recurrence rate at eight years which was 7% lower for the treatment arm (24.3% versus 17.3%, p < 0.001), and a cumulative mortality rate 3.8% lower (11.8% versus 7.8%, p = 0.005). Restricting the data to the N 0/1 patients, the benefit persists in the reduction of recurrence (19.4% versus 12.7%, p = 0.005) and the mortality rate at eight years (7.4% versus 5.3%, p = 0.12), although the latter is not statistically significant. The data for the HR- patient cohort is similar, such that the trastuzumab arm showed a recurrence and mortality rate at eight years which was lower than the arm without the treatment (33.4% versus 24%, p < 0.001 and 21.2 versus 12.4%, p = 0.001 respectively). In this meta-analysis, it is important to point out that the vast majority of the patients had T1c tumours and lymph node involvement. Similar benefits were found independently of HR, however the pattern of recurrence seemed to be different according to the HR expression, with the HR- subgroup having a higher risk of early relapse [30].

Globally these studies suggest that although these tumours generally have a very good prognosis without treatment and, in fact, in the majority of cases favourable outcomes can be expected without cytostatic or anti-HER-2 therapy, there are some patients who may benefit from treatment with chemotherapy and trastuzumab. As anticipated, classic factors for prognosis such as HR status and size seem to have an impact on the prognosis and may inform the treatment decision. For these patients, a cautious balance of benefits and toxicity should be weighed. The absolute benefit in the majority of cases was less than 5% in the reduction of the risk of distant recurrence. This is in fact the ideal context for developing less intense and toxic treatments.

A recent single-arm, multicentric study conducted by the Dana-Farber Cancer Institute assessed the benefits of chemotherapy associated with trastuzumab in HER-2+ patients with tumours less than or equal to 3 cm without lymph node involvement. DFS was the primary outcome. The treatment regimen consisted of weekly paclitaxel - 80 mg/m^2^ - for 12 weeks associated with 3-weekly trastuzumab which was continued for one year of treatment (TH regimen). Nearly 50% of a total of the 406 patients recruited had tumours with 1 cm or less and 91% of these had tumours with 2 cm or less. The results obtained after 3.6 years of follow-up showed a DFS of 98.7% (CI 95%, 97.6–99.8; p < 0.01) [31]. The data from this study was practice changing, leading to an update on several guidelines, such as the NCCN guidelines and the St Gallen consensus which now recommend this regimen for patients with small node negative HER-2+ tumours (Table 3).

## 4 Toxicity associated with systemic treatment

When we think about adjuvant treatment of breast cancer, it is fundamental that the benefits outweigh the risks of severe toxicity in the short and long term. Trastuzumab related cardiotoxicity presents most frequently with an asymptomatic reduction in the left ventricular ejection fraction, and rarely with symptomatic congestive heart failure. In contrast to anthracyclines, this does not seem to be related to the cumulative dose as it is normally reversible with the discontinuation of treatment, which permits an attempt to reintroduce the drug following recovery [32–34].

Trastuzumab is associated with a low incidence of severe (grade III or IV) cardiotoxicity with a reported incidence among the several studies which can reach 4%, when trastuzumab was used, against 1.3% in regimens which did not use this drug. In a meta-analysis of eight studies which included 11,991 patients, the use of trastuzumab was associated with an increase in the risk of cardiac failure by five times and reduction in the left ventricular ejection fraction by about two times [35]. On the other hand, the incidence of class III and IV cardiac failure seems to be significantly lower in the patients who did not receive anthracyclines as shown in the BCIRG 006 - 0.4% for the TCH (docetaxel, carboplatin, and trastuzumab) regimen versus 2–4% for those who received anthracyclines (AC followed by paclitaxel and trastuzumab).

The patients included in the trastuzumab adjuvant studies were relatively young and cardiac disease was an exclusion criteria. The risk of cardiotoxicity for the general population is therefore greater [36–39]. For example, in a retrospective analysis including 442 patients treated with anthracyclines and trastuzumab, the cumulative incidence of cardiac failure/cardiomyopathy reached 20.1% in five years [37]. In addition to cardiotoxicity, other toxicities related to chemotherapy such as neuropathy, leukaemia, and death should also be considered as well as the non-negligible risk of hospitalisation in the context of chemotherapy [40].

The TH regimen showed an excellent safety profile in the study by Tolaney *et al*. Only two patients (0.5%) showed grade III cardiotoxicity (cardiac failure), which was reversed in both following the interruption of the treatment with trastuzumab. Another 13 patients (3.5%) developed an asymptomatic reduction of the ejection fraction with recovery to normal levels in 11 of these cases. No death related to the treatment was reported [31].

Clinical trials which identify regimens with a more favorable toxicity profile are necessary in order to maximize the cost-benefit ratio of adjuvant treatments in this subgroup of patients. Clinical trials such at ATEMPT (TDM1 versus paclitaxel/trastuzumab for breast; NCT01853748) and RESPECT (assessment of trastuzumab without chemotherapy as a postoperative adjuvant treatment for breast cancer in HER-2+ patients; NCT01104935) are examples of trials exploring the activity of less toxic regimens. Other strategies, such as dual anti-HER-2 blocking or anti-HER-2 agents associated with endocrine treatment are being evaluated.

## 5 Discussion

Since 2005, several randomized multicentric prospective clinical trials have established trastuzumab in combination with chemotherapy as the standard treatment for HER-2+ early breast cancer, demonstrating substantial gains in DFS and OS [11–15]. Nonetheless, most of these studies included patients with positive lymph nodes, and the majority excluded infracentrimetric tumours. The data derived from these studies was analyzed in a 2007 meta-analysis, which demonstrated a reduction of approximately 50% in the risk of recurrence and mortality, independent of lymph node status [41]. In addition, the benefit from trastuzumab was observed in all stages. In a subgroup analysis, T1c tumours without lymph node involvement showed benefits of the same magnitude in relation to the other high-risk groups from the addition of trastuzumab to adjuvant chemotherapy [12, 13, 42]. Thus, assuming a 50% reduction in the risk of recurrence using chemotherapy plus trastuzumab for the T1ab population, the benefit of treatment would be directly related to the absolute risk of recurrence.

**Figure 1.**
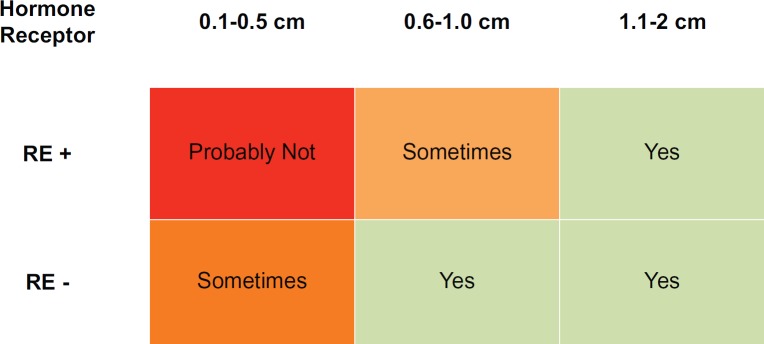
Suggestion for adjuvant therapy in Small Her-2+ Breast Cancer. Legend: HR+: Hormone receptor positive, HR-: Hormone receptor negative.

In the studies presented in this article, a risk of recurrence which varies with size and HR is currently estimated. Here we reviewed several papers which suggest that size and HR also play a role in the risk of recurrence among patients with infracentimetric tumours. In addition, HR status seems to differentiate between two distinct subgroups in terms of the risk of recurrence. Thus, and according to the recommendations of the NCCN, there will be patients with tumours of less than 1 cm (in addition to those with larger tumours and HR-) who can benefit from chemotherapy plus trastuzumab. Figure 1 shows a possible treatment algorithm for these patients.

The recently published prospective study by Tolaney *et al* shows that in a cohort of patients with HER-2+ tumours of up to 3 cm, the survival rate at three years following treatment with the TH regimen was excellent with a favorable toxicity profile [31]. Therefore TH seems the best regimen to be used among patients with infracentimetric HER2+ tumours who we decide to treat.

The decision to prescribe adjuvant treatment based on trastuzumab should then weigh the toxicities inherent to this treatment, seeking to minimise the risks, and the possible absolute benefit deriving from this treatment for this population which has a low risk of recurrence. Prospective studies are difficult to develop and recruit in this scenario, thus it is essential to clarify the actual benefit for these patients.

## Conclusion

Overall, the prognosis of patients with small (<1 cm) HER-2+ breast cancer is excellent. However in some selected cases the use of chemotherapy and/or targeted therapy may provide additional benefit. The therapeutic decision should always include a careful assessment of the risk-benefit ratio. Classical prognostic factors (e.g., size, nodal status, hormone receptor status) matter even in the setting of small tumours. Since this is a population with high survival rates the absolute benefit of any additional systemic therapy will always be marginal. In this context, the major challenge in the near future is to develop less toxic treatment regimens.

## Conflict of interest

All of the authors agree with the publication of the article and declare that there are no conflicts of interest of any kind.

## Figures and Tables

**Table 1.** Role of HER-2 in the prognosis for breast cancers of ≤1 cm with negative lymph nodes.

Study	Population	HER+ (% of population)	Monitoring (years)	Adjuvant CT/HT	Outcome for the study population (HER-2+ versus HER-)
Press *et al* 1997 N = 242 (23)	N0 (22.7% T1ab)	46 (19%)	6.8	No adjuvant treatment	RFS at two years: 83 versus 94% (p < 0.05)
Joensuu *et al* 2003 N = 852 (21)	T1abc (36.7% T1ab)	69 (12%)	9.5	1% (CMF)/4%	DDFS at nine years: 73 versus 89% (p = 0.0003)
Tovey *et al* 2009 N = 362 (24)	T1–2 G1–2	22 (6%)	6.5	9%/82%	BCSS at five years: 68 versus 96% (p < 0.001)
Chia *et al* 2008 N = 2026 (8)	T1–3 (210–16% T1ab)	206 (10.2%)	12.4	11%/20%	RFS at ten years 65.9 versus 75.5% (p = 0.01)
Curigliano *et al* 2009 N = 2130 (25)	T1ab	150 (7%)	4.6	27%/59%	DFS at five years: RE+: 92 versus 99% (p = 0.013) RE-: 91 versus 92% (p = 0.09)
Gonzalez-Ângulo *et al* 2009 N = 963 (9)	T1ab	98 (10.1%)	6.2	0/55%	RFS at five years 77.1% versus 93.7% (p < 0.001)
Fehrenbacher *et al* 2014 N = 234 (22)	T1ab	234 (100%)	5.8	47.9%/25.6%	RFI at five years: α T1a: 97.4% T1b: 90.9%
Vaz-Luis *et al* 2014 N = 4113 (10)	T1ab	528 (12.8%)	5.5	75.5%/17.1%	IDFS at five years:^*^ T1a: 84–86% versus 86–93% T1b: 68–86% versus 81–91% DRFS at five years:^*^ T1a: 93–96% versus 93–98% T1b: 94% versus 90–96%

α Data available only for HER-2+ tumours

* Population which did not receive adjuvant trastuzumab/chemotherapy

Legend: BCSS = Breast cancer specific survival, DDFS = Distant disease-free survival, IDFS = Invasive disease-free survival;

RFI = Recurrence-free interval; RFS = Recurrence-free survival; ER+ = Oestrogen receptor positive; ER- = Oestrogen receptor negative; T = Trastuzumab; CT = Chemotherapy; N = Number of patients; N0 = Negative lymph nodes, T1ab: ≤1 cm;

CMF: Cyclophosphamide, methotrexate, 5-fluorouracil.

**Table 2.** Outcomes by treatment for T1abN0 HER2+ tumours.

	Outcome	Without trastuzumab				With trastuzumab			
Rodrigues *et al* 2010 (29) N = 97	RFS at two years	94%				100%			
McArthur *et al* 2010 (28) N = 261	RFS at two years	93%				99%			
		**Without treatment**				**CT and/or T**			
		T1a		T1b		T1a		T1b	
Fehrenbacher *et al* 2014 (22)	RFI (five years)	97%		91.9%		100%		QT without T : 87.3% T with or without CT : 100%	
N = 234		100		71		16		47	
Ines-Vaz *et al* 2014^*^Δ (10)		RE-	RE+	RE-	RE+	RE-	RE+	RE-	RE+
N = 520		49	102	17	89	32	33	88	110
	DDFS (five years)	93%	96%	94%	94%	100%	100%	94%	96%
	IDFS (five years)	84%	86%	68%	86%	89%	100%	94%	90%

* T1a: 70.7% and 95.3% received trastuzumab and chemotherapy, respectively

Δ T1b: 60.5% and 98.4% received trastuzumab and chemotherapy, respectively

Legend: DDFS = Distant disease free survival, IDFS = Invasive disease free survival; RFS = Recurrence free survival; ER+ = Oestrogen receptor positive; ER- = Oestrogen receptor negative; T = Trastuzumab; CT = Chemotherapy; N = Number of patients.

**Table 3.** Prospective studies for HER-2+ breast tumours.

Study Characteristics				Population			Outcomes	
Study	N/CT Model	Phase	Monitoring (years)	Inclusion	% LFN negative	% < 1 cm	DFS	ICC*
N9831 + B-31 Perez *et al*, 2011 (12)	A: 2028/AC-TH B: 2017/AC- T	III	3.9	>1 cm if ER - >2 cm if ER +	5.7%	0%	A: 85.8% (four years) B: 75.8%	3.3–3.8%
HERA Goldhirsch *et al*, 2013 (43)	A: 1552/AC-TH B: 1697/AC-T	III	8	>1 cm if LFN negative Or positive lymph nodes	32.5%	NR	85.8% (two years)	1.7%
BCIRG-006 Slamon *et al,* 2011 (15)	A: 1073/ACT B: 1074/AC-TH C: 1075/TCH	III	5.4	Any size	28.6%	4.6%	84% (five years) 93% if T < 1 cm (ACTH)	2% (up to 4%)
Tolaney *et al*, 2015 (31)	**406/TH**	**II**	**3.6 years**	**≤3 cm** **(50% ≤ 1 cm)**	**~99%** **(six patients with N1mi)**	**50%**	**98.7%** **(three years)**	**0.5%**

Legend: AC: Doxorubicin, cyclophosphamide; TH: Concomitant taxane, trastuzumab; TH: Sequential; CCI: Congestive cardiac insufficiency (Grades III and IV); LFN = Lymph nodes; DFS: Disease free survival; CT = Chemotherapy; N1mi = Microscopic involvement;

NR: Not reported; ER+ = Oestrogen receptor positive; ER- = Oestrogen receptor negative.
